# The Role of mRNA Alternative Processing in Mammalian Neurodevelopment

**DOI:** 10.3390/ijms262211075

**Published:** 2025-11-16

**Authors:** Xian Liu, Jian Zuo, Guicheng Zhang, Xiaoyu Han, Yao Tian

**Affiliations:** School of Life Science and Technology, The Key Laboratory of Developmental Genes and Human Disease, Southeast University, 2 Dongda Road, Nanjing 210031, China; 101300005@seu.edu.cn (X.L.); zuoj9625@163.com (J.Z.); 220233957@seu.edu.cn (G.Z.); m15725436177@163.com (X.H.)

**Keywords:** neurodevelopment, alternative splicing, alternative polyadenylation, RNA chemical modification, neurodevelopmental disorder

## Abstract

The mammalian brain undergoes a series of orderly developmental events, including neurogenesis, neuronal migration, axon guidance, and synaptic connection. These neurodevelopmental mechanisms have traditionally been characterized through studies focused on transcriptional control; however, a growing body of evidence highlights the critical roles of co- or post-transcriptional steps like alternative splicing, alternative polyadenylation, and RNA chemical modification in orchestrating brain development. This review discusses the recent progress made toward understanding the influence of alternative mRNA processing on neurodevelopment, including three aspects: the key mRNA processing events that drive neuronal differentiation from stem/progenitor cells; the regulatory mechanisms that govern cell-type and stage-specific mRNA-processing patterns; and the neuropathological consequences of mRNA-processing dysregulation. By integrating these insights, we aim to deepen the understanding of how mRNA alternative processing influences neurodevelopment and its implications for neurological health.

## 1. Introduction

In eukaryotic organisms, precursor mRNA (pre-mRNA) molecules transcribed by RNA polymerase II require precise co- and post-transcriptional processing to generate mature mRNAs. Alternative mRNA processing substantially increases transcriptome diversity, which alters coding information [1,2], interaction domains [3,4,5,6], RNA stability [5,7], and subcellular localization [2,8], thereby affecting the specialization and functional state of cell types. This regulatory paradigm is particularly pronounced in the mammalian central nervous system (CNS), where the developmental trajectory demands exquisitely timed molecular programs to coordinate neurogenesis, neuronal migration, and synaptogenesis [9].

Pre-mRNA processing is primarily executed by the spliceosome and the cleavage and polyadenylation complex. The alternative splicing (AS) of pre-mRNAs occurs in approximately 95% of human genes and can alter coding information through skipped exons, retained introns, alternative 5′ or 3′ splice sites, or alternative first or last exons [10]. Developmentally regulated AS switches that generate critical isoform transitions are the fundamental determinant of cell-type specification [11]. Additionally, approximately 79% of mammalian genes undergo alternative polyadenylation (APA), generating isoforms that differ in coding sequence or 3′ untranslated regions (3′-UTRs). These differences can regulate RNA stability, localization, and translation efficiency [12,13]. Genome-wide RNA sequencing (RNA-seq) studies have revealed that stem/progenitor cells and differentiated cells exhibit different APA profiles. Proliferating neural progenitors preferentially utilize proximal polyadenylation sites (PASs) that generate the shorter 3′-UTRs, while differentiating neurons shift towards the distal PASs to generate the longer isoforms with extended regulatory sequences [12,14,15]. These observations suggest that APA is closely associated with neurogenesis. Furthermore, a growing body of research highlights the emerging roles of RNA chemical modifications, such as N6-methyladenosine (m^6^A) and 5-methylcytosine (m^5^C), in establishing dynamic post-transcriptional regulatory codes [16,17,18]. These modifications act as dynamic signals that inform cellular machinery about the status, location, and function of mRNA molecules, ensuring the precise orchestration of gene-expression programs essential for brain development.

In this review, we will discuss how mRNA-processing events, including AS, APA and RNA modification, influence neural development, with a particular focus on the following three aspects: (1) cell-specific mRNA-processing programs that drive neuronal differentiation from progenitor cells; (2) the regulatory mechanisms underlying cell-type or developmental-stage specific mRNA processing; and (3) the neuropathological consequences of mRNA-processing dysregulation. By delineating these mechanisms, we aim to shed light on the critical role of mRNA processing in shaping the neurodevelopmental landscape and its implications for neurological health and disease.

## 2. Alternative Splicing

**Figure 1:**
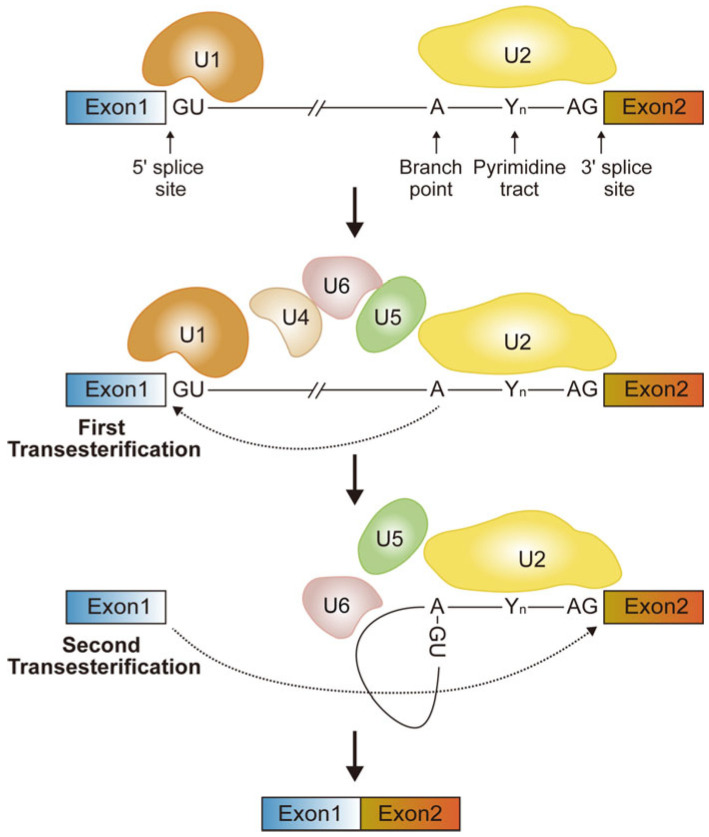
Fundamental mechanism for splicing. Initially, U1 snRNP binds to the 5′ splice site, and U2 snRNP binds to the branch point sequence (containing the critical adenosine, A), facilitated by the U2AF protein, which associates with the pyrimidine tract (Yn) and the 3′ splice site (AG). U1 and U2 are joined by the snRNPs U5 and U4–U6 complexes to form the precatalytic spliceosome. Next, U4–U6 complexes unwind, releasing U4 and U1. U6 pairs with the 5′ splice site, cleaving it and forming an intron lariat. In the second step, U5 aligns the splice sites, and the upstream exon’s 3′ OH-group joins with the downstream exon, excising the intron as a lariat. The spliceosome disassembles, recycling its components.

Splicing is a crucial step of post-transcriptional gene expression that is executed by the spliceosome machinery, comprising five evolutionarily conserved small nuclear ribonucleoproteins (U1, U2, U4, U5, U6 snRNPs) and over 150 auxiliary regulatory proteins, which are responsible for the precise removal of non-coding introns through a two-step transesterification reaction [19,20] (Figure 1). Beyond the basic process of constitutive splicing, AS serves as a key mechanism for expanding the transcriptomic and proteomic diversity of eukaryotic cells by selectively including or excluding specific exonic/intronic sequences through seven principal modes: (1) alternative 5′ terminal exons; (2) alternative 3′ terminal exons; (3) alternative 5′ splice sites [A5SS]; (4) alternative 3′ splice sites [A3SS]; (5) skipped exons [SE]; (6) mutually exclusive exons [MXE]; and (7) retained introns [RI] [10]. Among these modes, exon-skipping events are the most common, accounting for approximately 38% of AS events in human genes [21].

### 2.1. The Role of Alternative Splicing in Brain Development

Transcriptome profiling demonstrated a higher prevalence of AS events in brain compared to other tissues, with these events often occurring in a cell-specific or developmental-stage-specific manner [22,23]. A comprehensive analysis of human cerebral organoids and fetal neocortex revealed different splicing patterns in intermediate progenitor cells, radial glial cells, immature neurons, and neurons during cortical development [24]. A transcriptome analysis for the mouse cortex across nine developmental stages revealed over 20,000 distinct AS events between each stage, with the exon-skipping events predominating over exon inclusion during the developmental process [25]. Recent technological breakthroughs in single-cell and spatial long-read sequencing bring new insights into the cellular isoform diversity in the brain [26,27,28]. By using an enhanced single-cell long-read method (ScISOr-Seq2), Tilgner, H and colleagues generated the first comprehensive mRNA isoform atlas of the developing and adult mouse brain and discovered an unappreciated degree of isoform variability across multiple axes, further demonstrating the dynamic nature of AS events in different cell types, brain regions, and developmental stages [29].

Beyond cataloging AS events, a growing body of functional studies has begun to elucidate how specific splicing isoforms directly influence neuronal development and function. In NPCs, NUMB, as a critical effector of Notch signaling, is involved in asymmetric cell division and fate determination. The mammalian *Numb* gene produces at least four major isoforms through the alternative inclusion or skipping of exon 3 and exon 9. Functional rescue experiments in a *Numb*-null background revealed that the exon 9-included isoforms (NUMB1 and NUMB3) suppress neuronal differentiation and expand the progenitor pool, whereas the exon 9-skipped isoforms (NUMB2 and NUMB4) promote a reduction in proliferating progenitors, underscoring distinct functions of exon 9-included and exon 9-skipped NUMB in development and differentiation [30,31].

During axonogenesis, the splicing switch of *Shtn1* coordinates axon specification and growth. The long isoform, SHTN1L (exons 15 and 16-included), is predominantly expressed during early corticogenesis, while the short isoform, SHTN1S (exons 15 and 16-skipped), becomes dominant postnatally when primary axon formation is completed. Inagaki’s group reported that SHTN1S overexpression induced multiple short axons, while overexpressing SHTN1L did not lead to an increase in axon number [32,33]. Further studies have indicated that the specific depletion of SHTN1L in neurons decreased axon length without affecting neurite number [34]. These findings indicate that SHTN1S promotes axon specification, while SHTN1L is necessary for axon elongation.

Cell-type-specific AS also creates molecular diversity in synaptic proteins. Notably, neurexins (NRXNs) are a family of presynaptic cell adhesion molecules, including NRXN1, 2, and 3. Thousands of NRXN isoforms can be produced through AS at six canonical sites (SS1–SS6), with the SS4 site being particularly well-studied [4,35]. The inclusion of the SS4 exon strongly influences NRXN’s binding affinity for postsynaptic partners such as neuroligins and CBLN1-GLUD2 complexes. Moreover, in mouse hippocampal neurons, AS at the presynaptic *Nrxn1* SS4 site impacts excitatory postsynaptic currents mediated by ionotropic glutamate receptor NMDAR and influences spatial learning and memory in mice. When NRXN1 SS4(+) isoforms are expressed, there is a significant increase in postsynaptic NMDAR response, whereas with the expression of NRXN3 SS4(+) isoforms, there is a notable enhancement in postsynaptic AMPAR response, suggesting that a single alternative exon is essential for circuit function and behavior [36,37,38,39,40].

This intricate splicing landscape highlights AS is a fundamental regulatory mechanism in neural development, and its extensive and dynamic nature is determined by the developmental stages, cell types, and brain regions.

### 2.2. The Timely Expression of RBPs Regulates the AS Switch in Neural Development

Due to the fact that most of the spliceosome components are ubiquitously expressed, the dynamic regulation of AS during neural development is predominantly orchestrated by RNA-binding proteins (RBPs), which exhibit developmental-stage-specific and tissue-specific expression patterns [41,42,43,44]. Below, we exemplify how the developmental-stage-specific RBPs influence splicing programs during neurodevelopment.

**Figure 2:**
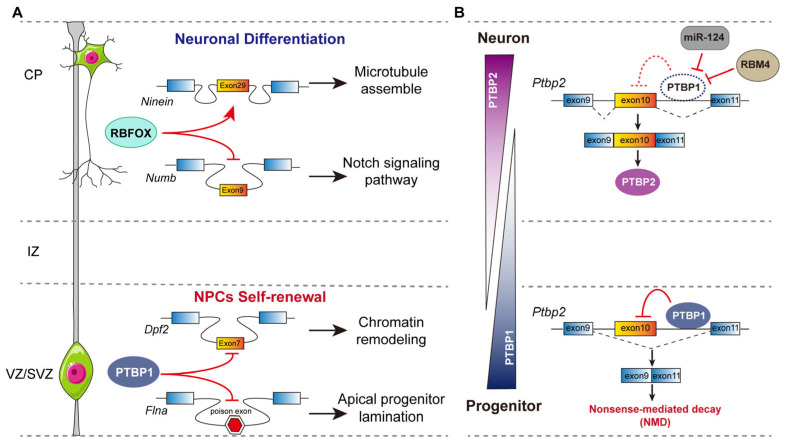
The role of selective RBPs in neurogenesis. (**A**) Cell-type-specific AS regulated by RBFOX and PTBP1 proteins governs neural progenitor cell differentiation. PTBP1 is expressed in NPCs (yellow, VZ/SVZ) and represses the neuronal exon (*Dpf2*) and poison exon (*Flna*) inclusion to maintain NPC self-renewal, while RBFOX1/2/3 proteins are highly expressed in neurons (blue, CP) to promote neuronal exon inclusion (*Ninein*) and NPC exon exclusion (*Numb*). (**B**) Sequential functions of the mammalian paralogs PTBP1 and PTBP2 are mediated by their cross-regulation. In progenitors, PTBP1 promotes the skipping of exon 10 in *Ptbp2* pre-mRNA, produces an RNA substrate for nonsense-mediated decay (NMD). In neurons, the increased RPM4 and neural-specific miR-124 down-regulate PTBP1, thereby derepressing the exon 10 of *Ptbp2*, allowing for PTBP2 expression.

The PTBP family, comprising PTBP1, PTBP2, and PTBP3, shares structural homology and RNA-binding preferences but exhibits distinct expression patterns. PTBP1 and PTBP2 play important roles in neuronal development, whereas PTBP3 is not involved in this process [42,45]. PTBP1 is highly expressed in neural stem cells (NSCs) and progenitors, but is downregulated as NPCs differentiate into neurons with a concomitant increase in PTBP2 levels [46]. This change in regulators induces changes in splicing of a large set of alternative exons, driving the transition from neural stem/progenitor cells (NSPCs) to neurons [46,47]. For example, PTBP1 controls splicing of *Dpf2*, a subunit of BRG1/BRM-associated factor (BAF) chromatin-remodeling complexes. *Dpf2* exon 7 is inhibited by PTBP1 to produce the DPF2-S isoform early in development. During neuronal differentiation, loss of PTBP1 facilitates exon 7 inclusion and DPF2-L expression. This AS switch redirects BAF complex targeting to impact chromatin organization during neuronal development [48]. Moreover, PTBP1 represses the inclusion of poison exons within *Flna* and *Flnb* transcripts in NPCs. Its depletion reduces FLNA/FLNB protein levels, leading to NPC detachment from the ventricular zone, premature differentiation, and lethal hydrocephalus [49,50] (Figure 2A). PTBP2-null brain exhibited an aberrant progenitor polarity, premature neurogenesis, and reduced progenitor pools [51]. Actually, PTBP1/PTBP2 transition is executed by a mutual exclusion mechanism: PTBP1 promotes the skipping of exon 10 in *Ptbp2* pre-mRNA, introducing a premature termination codon that triggers nonsense-mediated mRNA decay (NMD), thereby repressing PTBP2 expression in PTBP1-expressing cells. As the expression of PTBP1 diminishes during neuronal differentiation, exon 10 of *Ptbp2* is derepressed, allowing for PTBP2 expression, which is essential for neuronal maturation [45,52,53] (Figure 2B).

SRRM4, a conserved SR protein in vertebrates, also known as nSR100, is another critical regulator of AS during neural development. In mammalian cell culture and *zebrafish* models, SRRM4 has been shown to be required for neurogenesis and neuronal differentiation [54,55]. Moreover, the expression of SRRM4 increases upon neuronal maturation [56], and it can regulate the AS of neural microexons, ranging from 3 to 27 nucleotides in length. Strikingly, many microexons display a “switch-like” inclusion or exclusion during neuronal maturation. These microexon AS events are implicated in the regulation of protein interaction networks during neuronal development, influencing neuronal cell-type specification and neural circuit formation [57]. Knockout of SRRM4 in mice causes premature neuronal differentiation of NPCs, resulting in defective cortical layering and the impaired midline crossing of callosal axons, alongside reduced neurite outgrowth [57].

The RBFOX family, encompassing RBFOX1, RBFOX2, and RBFOX3, represents another essential group of RBPs in neural development. RBFOX1 and RBFOX2 are widely expressed in neurons, skeletal muscle, and cardiac muscle, while RBFOX3 expression is restricted to post-mitotic neurons [58]. All three RBFOX genes have been shown to generate alternatively spliced variants of coding sequence in mammals, producing a full-length isoform and a truncated-splice isoform lacking an RNA-recognition motif. The splicing switch of RBFOX3 from the truncated isoform to the full-length isoform occurs in a development-dependent manner, and the latter generally promotes the inclusion of many neuronal exons [59]. Moreover, genome-wide studies have demonstrated that RBFOX proteins directly control the splicing of genes that are upregulated during neurodevelopment and linked to autism spectrum disorders (ASD) [60,61]. Studies have established various RBFOX knockout mouse models to investigate its function in neurodevelopment. While the isolated knockout of RBFOX1 or RBFOX2 causes minimal cortical malformation due to functional redundancy, RBFOX1 KO mice exhibit increased seizure susceptibility, and RBFOX2 KO leads to cerebellar defects and ataxia [62,63]. RBFOX3 is required for late neuronal differentiation; its depletion impairs cell fate determination through *Numb* mis-splicing [61]. All three RBFOX proteins promote neuronal differentiation by favoring the inclusion of a neuronal exon in the *Ninein* transcript [50] (Figure 2A). Moreover, triple knockout of RBFOX1/2/3 in motor neurons impairs excitability and disrupts axon initial segment assembly via the defective splicing of Ankyrin G [64].

The neuro-oncological ventral antigens NOVA1 and NOVA2 are neuron-specific RNA-binding proteins that function as key regulators of AS, influencing neuronal differentiation and synaptic connectivity. NOVA1 and NOVA2 exhibit distinct spatiotemporal expression profiles: NOVA2 is predominantly expressed in the cortex, hypothalamus, and striatum, whereas NOVA1 is more abundant in the cerebellum and ventral spinal cord [65,66]. Structurally, both NOVA proteins possess three KH homology (KH) domains that bind to YCAY motifs in pre-mRNAs, typically located near exon–intron boundaries [67,68]. The functional outcome of NOVA binding—whether it promotes inclusion or skipping of an exon—depends on the location of these YCAY elements relative to the regulated exon. Binding within or upstream of an exon generally leads to exon skipping by hindering U1 snRNP recruitment, whereas binding downstream tends to enhance exon inclusion by facilitating spliceosome assembly [69]. By targeting synaptic genes such as the GABAA receptor γ2, glycine receptor α2 subunits, NOVA-mediated splicing directly regulates synaptogenesis, synaptic vesicle release, and neurotransmitter signaling [41,70]. Beyond these shared functions, NOVA2 uniquely governs AS programs essential for axon guidance. In the absence of NOVA2, mis-splicing occurs in key axonal pathfinding genes such as *Neo1*, *Dcc*, *Robo2*, and *Slit2*. These disruptions are thought to underlie the defective commissural axon projections observed in NOVA2-deficient models [70,71,72].

Splicing regulators function as part of an intricate, interconnected molecular network rather than operating independently. Rogalska et al. systematically depleted more than 300 spliceosome components or regulators, profiled the resultant AS changes, and further inferred their functional relationships. They have identified over 82,000 shared splicing alterations across all 305 knockdown groups, with each splicing factor’s depletion affecting the AS of an average of 24 other splicing factors. This underscores the extensive and intricate network of interactions among RBPs [73]. As mentioned earlier, PTBP1 and PTBP2 exemplify such regulatory crosstalk. PTBP1 represses PTBP2 expression by promoting the skipping of exon 10 in *Ptbp2* pre-mRNA to keep the cells undifferentiated. When differentiation begins, the microRNA (miR-124) binds the 3′-UTR of *Ptbp1* and rapidly lowers PTBP1 protein levels [53]. Although miR-124 can also bind *Ptbp2*, ELAVL3 blocks this site, shielding *Ptbp2* mRNA from degradation; PTBP2 levels therefore increase [74]. On the other hand, RBM4 is a ubiquitous RBP with an elevated level during neuronal differentiation, which regulates the splicing of exon 9/10 of *Ptbp1* and downregulates PTBP1 levels [75,76]. At the same time, the splicing factor SRRM acts as a positive modulator to promote inclusion of exon 10 in *Ptbp2*, producing the full-length PTBP2 protein that drives neuronal maturation [57,77]. This interconnected molecular network ensures the timely expression of PTBP2 as neural development progresses. Additionally, PTBP1 has been shown to inhibit AS events activated by SRRM4 in approximately 30% of cases, highlighting the antagonistic relationship between these factors [55]. Furthermore, PTBP1 and NOVA proteins [78], as well as PTBP1 and RBFOX proteins [79], exhibit antagonistic interactions, whereas RBFOX and NOVA may synergistically regulate specific splicing events [80].

These intricate regulatory networks among RBPs are crucial for establishing the spatial and temporal specificity of proteins, which in turn governs the complicated AS patterns observed in the nervous system. This coordinated regulation ensures the precise execution of splicing programs, which are essential for neuronal differentiation, maturation, and function.

## 3. Alternative Polyadenylation

**Figure 3:**
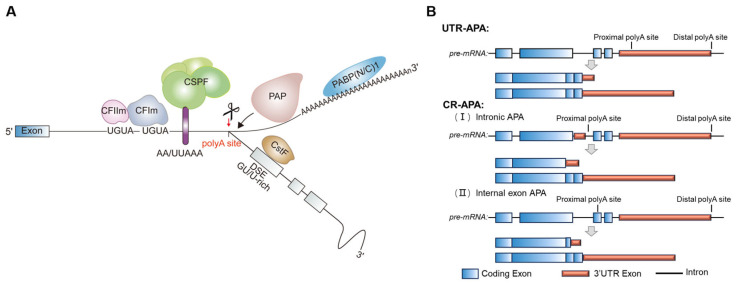
Cleavage and polyadenylation of mRNA isoforms at their 3′ ends. (**A**) Fundamental mechanism for polyadenylation. The multi-subunit cleavage and polyadenylation complex recognizes the pre-mRNA 3′ end via sequence motifs. Following cleavage at the polyA site (red arrow) by the CPSF73 endonuclease, polyA polymerase (PAP) binds to the 3′ end of pre-mRNA (black arrow) to generate the polyA tracts. (**B**) Schematic representation of UTR-APA and CR-APA. UTR-APA produces distinct mRNA isoforms with different-length 3′-UTRs but encodes the same protein. CR-APA, involving PASs within different terminal exons or introns, produces mRNA isoforms with distinct C-terminal coding regions, resulting in distinct protein isoforms. Red boxes indicate untranslated regions; light blue boxes indicate coding exons; and lines indicate introns.

In eukaryotic cells, most protein-coding transcripts undergo 3′ end processing via cleavage and polyadenylation (CPA), except for the canonical replication-dependent histone transcripts. This process involves endonucleolytic cleavage of nascent RNA followed by poly(A) tail synthesis by poly(A) polymerase. Over 79% of mammalian genes contain multiple PASs, allowing for the production of distinct mRNA isoforms through APA [81,82]. APA can be classified into two main types based on where the alternative PAS is located: (1) tandem 3′-UTR-APA (UTR-APA), which generates transcripts differing solely in 3′-UTR length that contain extended regulatory sequences, thereby modulating mRNA stability, translation efficiency, or subcellular localization [83,84]; and (2) coding region APA (CR-APA), involving PASs within alternative terminal exons, internal exons, or introns, leading to truncated protein variants [13,85,86] (Figure 3). The diverse APA configurations have been shown to impact gene expression at different levels.

### 3.1. Unique Landscape of APA Regulation in Neural Tissue

APA regulates many biological processes, such as embryonic development [15], tissue homeostasis [87], and immune responses [88], with particularly significant effects in the nervous system [89,90]. Early microarray studies have revealed that many genes in the human brain preferentially use distal PASs, producing transcripts with longer 3′-UTRs [10,91,92,93]. Subsequent advances in deep sequencing technologies have corroborated the widespread prevalence of 3′-UTR extension across human and mouse neural tissues, whereas the usages of proximal PASs are favored in blood cells and testis tissue [12,94,95,96]. This APA bias has also been observed in *Drosophila* and *zebrafish*, with CNS-specific 3′-UTR up to 20 times longer than in other tissues, showing its evolutionary conservation [97]. More intriguingly, emerging evidence establishes a dynamic correlation between APA patterns and neural cell state transitions. Studies by Shepard, Lackford, and others using 3′ end-sequencing technologies like PAS-seq and 3′READS have revealed that proliferating cells like neural stem cells/progenitors (NSPCs) preferentially use proximal PASs to generate the shorter transcripts, whereas differentiation initiation triggers a genome-wide shift toward the usage of the distal sites. This transition generates lengthened 3′-UTR isoforms enriched with *cis*-regulatory elements for post-transcriptional control. Remarkably, when mature neurons are reprogrammed back to a stem cell-like state, their APA pattern reverts to a preference for proximal sites [12,98,99,100]. This indicates that PAS selection during NSPC differentiation is subject to dynamic and directional regulation, serving as a key part of the gene-expression regulatory network to control the cellular functional states. In addition, the APA profile varies among distinct neuronal populations. Ying, W and colleagues characterized the dynamic APA profiles in different human neurons using single-nucleus 3′ RNA sequencing and observed a large number of APA switches between excitatory neurons and inhibitory neurons, suggesting APA may contribute to gene-expression regulation in specific cells, leading to neuronal heterogeneity [89].

APA is also involved in neuronal activation. A well-characterized example is brain-derived neurotrophic factor (*BDNF*), which generates a longer 3′-UTR isoform (BDNF-L) and a shorter 3′-UTR isoform (BDNF-S) through APA. BDNF-L is dramatically enriched in dendrites, whereas the BDNF-S is restricted to the soma. This distinct expression pattern controls the translation efficiency of BDNF under different neuronal activity [101]. Similarly, long and short isoforms of mRNAs encoding RanGTPase are localized to the axon and cell body, respectively [102,103]. These reports suggest that long isoforms are more likely to be located in dendrites or axons than are short isoforms. Conversely, the long isoform of CALM1, a key calcium-signaling integrator, is highly expressed in the soma of dorsal root ganglion (DRG) neurons, influencing DRG migration during embryonic development and experience-induced neuronal activation in the adult hippocampus [104].

Alternative 3′ UTRs modify the localization, stability, and translation efficiency of mRNAs in neuronal compartments. Tushev, Glock et al. used 3′ end sequencing combined with microdissection of hippocampal slices to separately profile transcripts from somata and neuropil. They revealed extensive diversity in neuronal 3′ UTRs, with isoforms encoding synaptic proteins exhibiting distinct localization, stability, and translation dynamics. Notably, the longer 3′ UTR isoforms localized to the neuropil were significantly longer lived than their shorter counterparts, supporting the notion that the longer half-life might help to establish localization to distal regions of the dendrite or axon [105]. Anthony G. Lau and colleagues directly tested the regulatory capacity of alternative 3′ UTRs from the BDNF gene using luciferase reporter constructs. They demonstrated that the long and short 3′ UTRs confer distinct translational control programs. Under resting conditions, the short 3′UTR, which is restricted to the neuronal soma, is predominantly associated with translating polyribosomes in the hippocampus and maintains basal levels of BDNF protein. Most importantly, upon neuronal activation, only the long 3′ UTR was sufficient to drive robust activity-dependent translation in both somatic and dendritic compartments [101]. This study provides direct functional validations that APA generates 3′ UTR isoforms, which serve as specialized regulatory modules to spatially and temporally control gene expression in response to neuronal activity.

### 3.2. The Regulatory Mechanism of APA in Neurodevelopment

The PAS is determined by the hexamer poly(A) signal (AAUAAA and its variants) and additional regulatory elements, including the UGUA enhancer upstream of the PAS (USE), U-rich elements surrounding the PAS, and U/UG-rich elements within approximately 100 nt downstream of the PAS (DSE). These sequence elements are specifically recognized by the multi-subunit protein complex and several single proteins. The cleavage and polyadenylation specificity factor (CPSF) binds to the AAUAAA hexamer, cleavage stimulatory factor (CstF) binds to the DSE, and cleavage factor Im (CFIm) recognizes the UGUA-containing USE. The assembly of these factors on RNAs is facilitated by the C-terminal domain of the RNA polymerase II (RNAP II), and they in turn recruit cleavage factor IIm (CFIIm) and poly(A) polymerase (PAP) to initiate cleavage and polyadenylation [106,107,108] (Figure 3).

**Figure 4:**
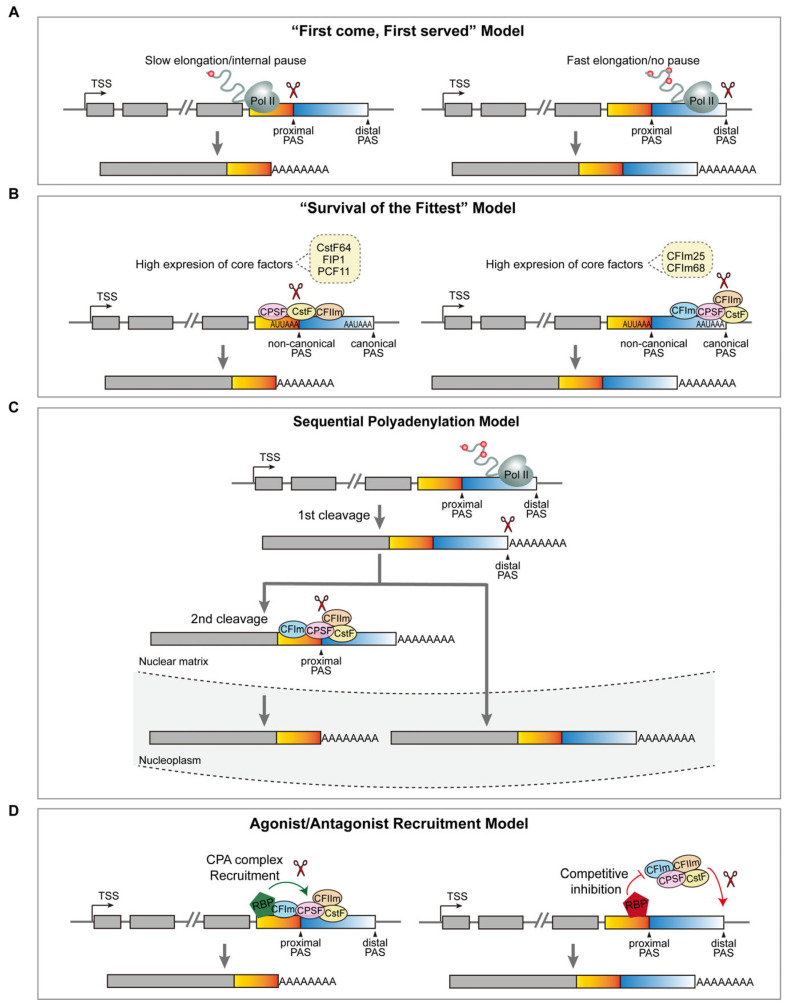
Various mechanisms for APA regulation. (**A**) “First come, first served” model. When the elongation rate is slow, the proximal site gains a significant temporal advantage over the distal site by being recognized earlier by 3′ end-processing factors, thereby increasing its likelihood of being selected. (**B**) “Survival of the fittest” model. The distal PAS is stronger than proximal sites due to more robust consensus motifs, and its selection is modulated by the concentration of 3′ end-processing factors. (**C**) Sequential polyadenylation model. CPA first occurs at an active distal PAS, resulting in transcripts with longer 3′UTRs. Subsequent CPA takes place at the proximal PAS, producing isoforms with shorter 3′UTRs. (**D**) Agonist/antagonist recruitment model. Regulatory factors can bind directly to sequences within or overlapping the core PAS. In the agonistic model (left), such binding facilitates the recruitment of the mRNA 3′ end processing machinery, thereby promoting the usage of the PAS. Conversely, in the antagonistic model (right), regulatory factors competitively inhibit the binding of the 3′ processing machinery to the PAS, which suppresses its usage and enhances the selection of the alternative PAS. TSS refers to the transcription start site. Yellow boxes indicate a shorter 3′-UTR; light blue boxes indicate an extended 3′-UTR; gray boxes indicate common exons; lines indicate introns.

Cell-type or developmental-stage-specific APA underscores the precision of PAS selection in distinct cellular contexts. Recent advances have elucidated four principal mechanistic frameworks governing APA dynamics (Figure 4): (1) First come, first served: Proximal PAS usage is inherently prioritized due to its earlier transcription compared to the distal PASs. This model posits that the distance between alternative PASs and the transcription machinery’s elongation rate is crucial for APA regulation [98,109,110]. This conclusion is indeed supported by the observation that a mutated RNAP II with a slower elongation rate displayed a significant shift in the PAS selection towards the proximal site. Furthermore, transcriptionally induced secondary structures (e.g., R-loops) promote RNAP II stalling between competing PASs, reinforcing proximal site utilization [111,112,113]. (2) Survival of the fittest: PAS selection is dictated by sequence-dependent signal strength, where proximal PASs often represent weak noncanonical variants, while distal PASs typically harbor stronger consensus motifs [98,114]. The equilibrium between these sites is modulated by the abundance of 3′ end-processing factors. For instance, the depletion of CstF64/CstF64τ or CPSF subunit Fip1 biases cleavage toward the stronger distal PASs, whereas reduced CFIm25/CFIm68 levels enhance the proximal PAS usage [98,114,115,116,117]. This model highlights the stoichiometric sensitivity of APA to core processing machinery. (3) Sequential polyadenylation model: While APA regulation is predominantly co-transcriptional, emerging evidence supports a post-transcriptional mechanism involving ordered PAS activation. In this model, CPA is first carried out at an active distal PAS, producing transcripts with a longer 3′-UTR. Subsequent CPA occurs at the proximal PAS and then produces shorter variants. This sequential polyadenylation expands the regulatory scope, allowing RBPs to influence APA outcomes at both transcriptional and post-transcriptional stages [118]. (4) Agonist/antagonist recruitment model: Beyond the core processing protein complexes, over 90 auxiliary regulators dynamically modulate APA by binding cis-elements either overlapping or adjacent to PAS sequences [119]. These factors act as molecular switches, facilitating or obstructing the recruitment of 3′ processing machinery to RNA substrates. Such agonist/antagonist interactions create a context-dependent regulatory layer, enabling fine-tuned PAS selection in response to cellular cues.

**Figure 5:**
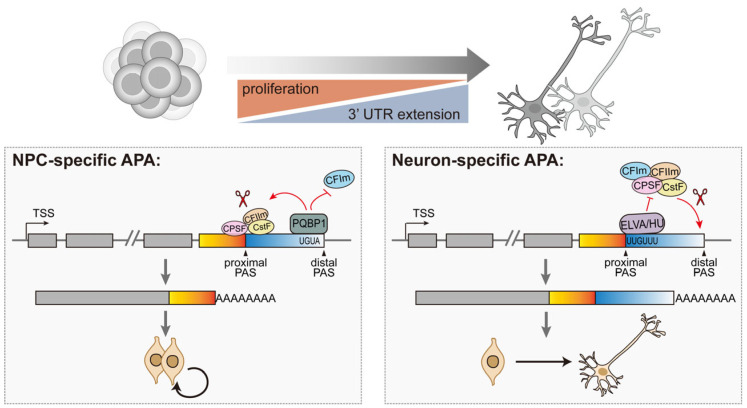
A proposed model of dynamic regulation of APA in NPCs and neurons. Differentiation of NPCs is accompanied by 3′-UTR lengthening. PQBP1 is highly expressed in NPCs and binds the distal UGUA motifs to block CFIm complex recruitment, which reduces cleavage at the distal sites and increases the proximal sites usage in cell cycle-related genes, thereby enhancing NPC proliferation. ELAV/HU proteins, enriched in neurons, bind preferentially downstream of proximal PAS and suppress CPA complex recruitment. Consequently, there is a global shift towards the generation of extended 3′-UTR isoforms in neural cells, promoting neuronal differentiation.

While accumulating evidence suggests that APA regulation is coupled to the expression levels of RBPs, current insights predominantly derive from experimental perturbations in pathological or gene-edited models [120]. For example, depletion of PCF11—a component of the CFIIm complex involved in transcription termination and RNA 3′ end maturation—in the murine central nervous system leads to pervasive dysregulation of APA and premature neurodifferentiation [121]. Similarly, loss of SAM68 results in the expression of a truncated ALDH1A3 isoform, altered cellular metabolism, and enhanced differentiation of NPCs, ultimately impairing cortical expansion [122]. Under physiological contexts, two RNA-binding proteins have been identified as molecular switches that govern dynamic APA during neurodevelopment (Figure 5): (1) neuronal-specific ELAV/HU family proteins, which bind to the upstream U-rich signal near the proximal PAS, hindering the recognition of the 3′ end-processing machinery and promoting the use of the distal PAS, resulting in the 3′-UTR elongation in neurons [123]. ELAV/Hu-deficient mice exhibit increased an self-renewal capacity regarding NPCs, though they generate fewer neurons [124]; (2) the intellectual-disability-associated protein PQBP1, which is enriched in NSPCs and binds the distal UGUA motifs to block CFIm complex recruitment. This antagonistic interaction favors the proximal PAS selection, generating shorter 3′-UTR isoforms and maintaining NSPC self-renewal. PQBP1-deficient progenitors exhibit aberrant usage of polyA sites within genes associated with the cell cycle, leading to decreased proliferation and enhanced differentiation [125].

However, both of the regulators exhibit sequence- and context-dependent specificity, targeting only subsets of genes with specific PAS architectures. Their limited regulatory scope implies the existence of additional RBP-mediated mechanisms orchestrating global APA dynamics during neurodevelopment. Supporting this hypothesis, single-cell transcriptomic profiling across five developmental stages and 38 neural cell types by Agarwal et al. has identified RBP families including RBFOX1-3 and CELF2-6 that exhibit monotonic expression increases during neural development [14]. Strikingly, their expression peaks in mature neurons and correlates temporally with the 3′-UTR elongation events, suggesting their potential roles in APA regulation, and the regulatory mechanisms remain to be elucidated in the future.

## 4. RNA Chemical Modifications

Over the past decade, accumulating evidence has shown that various mRNA chemical modifications can affect RNA metabolism in a cell-type and tissue-specific manner, giving rise to the field of epitranscriptomics. These modifications, which occur at specific nucleotide positions or structural regions, exert precise control over RNA metabolism by modulating mRNA stability, translational efficiency, subcellular localization, and degradation kinetics and consequently impacting multiple biological processes, including neurodevelopment, stress response, immune homeostasis, and tumorigenesis [126,127,128].

The advent of high-throughput sequencing technologies has catalyzed the discovery of over 170 distinct mRNA modifications at sites including the 5′ cap, exons, introns, and 3′-UTRs [126]. Methylation, the most prevalent category of mRNA modifications, involves the enzymatic transfer of a methyl group (–CH_3_) to nucleobases. Common forms of methylation include N6-methyladenosine (m^6^A) [129,130], 5-methylcytosine (m^5^C) [131], N1-methyladenosine (m^1^A) [132], N7-methylguanosine (m^7^G) [131], and 2′-O-methylation (Nm) [133]. Pseudouridine (Ψ) represents another major class of mRNA chemical modification, which alters the structure and function of mRNA by replacing the N-C bond with a new C-C bond, thus converting uridine (U) to Ψ [134,135]. In addition, some novel mRNA modifications have been identified in recent years. For instance, N4-acetylcytosine (ac4C), the sole acetylated RNA modification in eukaryotes, is catalyzed by N-acetyltransferase 10 (NAT10) at the N4 position of cytosine. This modification enhances mRNA-tRNA interactions in coding regions and regulates translation initiation in the 5′ UTR, underscoring its important role in mRNA translation [136].

**Figure 6:**
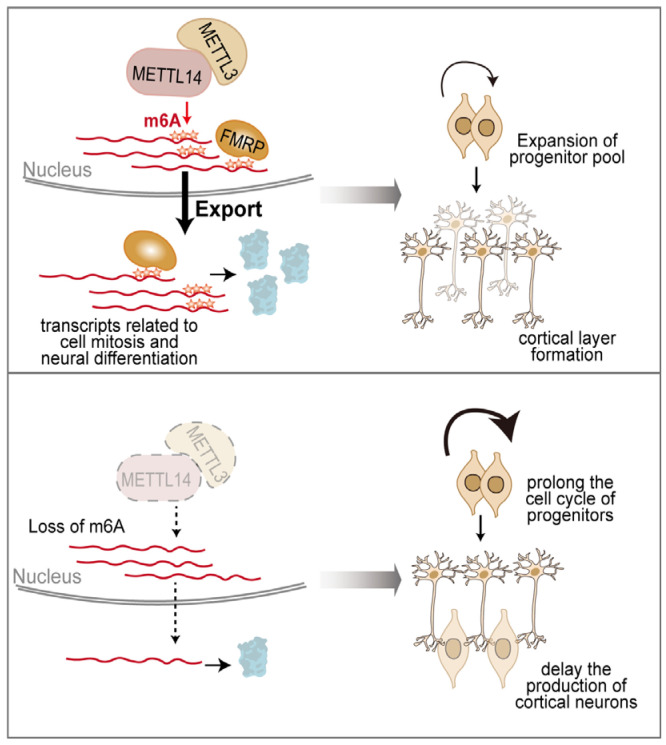
A model of how m^6^A modification regulates neurogenesis. m^6^A modification, catalyzed by writer proteins such as METTL3 and METTL14, is enriched in transcripts related to mitosis, stem cell maintenance, and neural differentiation and controls the proliferation and differentiation of NPCs. Decreasing m^6^A levels through the deletion of METTL3 or METTL14 prolongs the cell cycle of NPCs and delays the production of neuron subtypes of different cortical layers.

mRNA chemical modification is well documented as a vital post-transcriptional mechanism in the nervous system. m^6^A is the most abundant RNA modification, which is catalyzed by the methyltransferase complex consisting of METTL3, METTL4, WTAP, and KIAA1429, as well as RBM15 and its paralogue RBM15B, and displays dynamic features during embryonic and postnatal neuronal development [137]. Nearly half of all stably expressed genes in the adult mouse cortex carry m^6^A methylation, and perturbation of this modification triggers profound developmental defects in both the cerebral cortex and cerebellum [138,139,140]. m^6^A immunoprecipitation combined with deep sequencing (MeRIP-seq) has revealed that m^6^A is predominantly enriched in transcripts related to mitosis, stem cell maintenance, and neural differentiation, and controls both the proliferation and differentiation of NPCs [16,141,142]. Decreasing m^6^A levels through the deletion of METTL14 prolongs the cell cycle of cortical NPCs and delays the production of neuron subtypes of different cortical layers [141] (Figure 6). Similarly, depletion of METTL3 not only inhibits neuronal development and skews the differentiation of NSCs more toward glial lineage, but also affects the morphological maturation of newborn neurons in the adult brain through modulating Ezh2 [142]. More recently, VIRMA, a core and evolutionarily conserved component of the m^6^A methyltransferase complex, was shown to be required for forebrain development. Depletion of VIRMA destabilizes the m^6^A writer complex and reduces m^6^A levels, leading to decreased proliferation and increased apoptosis of NPCs, ultimately causing severe forebrain developmental defects [143]. m^6^A deposition could dynamically tune the stability and localization of the target RNAs through m^6^A readers. For example, the m^6^A reader YTHDF2 selectively binds m^6^A-modified transcripts and relocates them from the translatable pool to mRNA decay sites, such as processing bodies, thereby promoting their degradation. Deletion of YTHDF2 delays mouse neuronal development through impaired proliferation and differentiation of neural stem and progenitor cells [144]. FMRP preferentially binds m^6^A-modified RNAs to facilitate their nuclear export via the CRM1 pathway. Functional validation comes from integrated transcriptomic and m^6^A mapping analyses, which revealed that CKO of METTL14 (the m^6^A “writer”) and KO of FMRP both lead to the nuclear retention of a shared set of m^6^A-modified mRNAs and subsequently influence neural progenitor differentiation [145] (Figure 6). m^6^A modification can also modulate the localized mRNA translation in axons. During synaptic occurrences, m^6^A-modified transcripts are preferentially translated, ensuring the rapid synthesis of proteins crucial for cellular adaptation and synaptic transmission, further promoting memory formation, retention, and retrieval [17,146,147]. Local alterations of demethylase FTO in the developing brain mediate the dynamic gene expression associated with synaptic plasticity and memory formation [148,149].

RNA m^5^C, another extensively studied mRNA modification, is preferentially enriched downstream of translation-initiation sites and is broadly distributed across the whole coding sequence (CDS) of mammalian mRNAs. Transcriptome-wide bisulfite sequencing maps of RNA m^5^C have been generated for cultured NSCs, neurons, and mouse brains at key developmental stages. Neurons exhibit a markedly higher m^5^C density than NSCs, and the hypermethylated sites reside predominantly in the genes that are involved in positive transcriptional regulation and axon extension [18]. Moreover, the early postnatal brains display marked remodeling of both m^5^C landscapes and the expression of the corresponding writers, readers, and erasers [18]. The loss of RNA methyltransferase NSUN2 results in decreased m^5^C-tRNA levels, leading to the impairment of NSC differentiation and a decrease in upper-layer cortical neurons, and subsequently inducing microcephaly and motor defects [150,151,152]. In addition, the m^7^G modification, which is mainly found in the 5′ cap structure of mRNAs, recently gained considerable attention. Disruption of mRNA m^7^G modification in the adult mouse hippocampus can diminish neurogenesis and spatial memory [153]. These findings collectively demonstrate the crucial functions of RNA chemical modifications in brain development.

Although individual chemical modifications play indispensable roles in neurodevelopment, emerging epitranscriptomic studies have revealed that these modifications, such as m^6^A and m^5^C, form a highly coordinated regulatory network within the neuronal transcriptome [154]. Transcripts encoding m^5^C-associated effectors (e.g., NSUN4, NSUN5) have been found to harbor m^6^A modifications; meanwhile, the m^6^A effector transcripts (e.g., METTL3, YTHDF1) also carry m^5^C marks, establishing post-transcriptional feedback loops of reciprocal regulation. At the protein level, extensive co-regulatory relationships have been identified between m^6^A- and m^5^C-related factors. For instance, the m^6^A reader YTHDC2 functionally coordinates with the m^5^C writer NSUN2 in response to cellular perturbations, and the m^5^C reader ALYREF has been shown to physically interact with the components of the m^6^A writer complex, METTL14 and RBM15/RBM15B. In neurons, ALYREF exhibits cytoplasmic colocalization with m^6^A-modified RNAs, which significantly diminishes following NMDA receptor–mediated synaptic activation, indicating a spatially dynamic and activity-dependent association. These findings provide a molecular framework for how the m^6^A–m^5^C crosstalk contributes to neuronal maturation and synaptic plasticity [154]. Additionally, recent studies have also uncovered a dynamic interplay between m^6^A modification and ADAR-mediated adenosine-to-inosine (A-to-I) RNA editing. m^6^A can influence ADAR1 recruitment and editing efficiency in a position-dependent manner—enhancing ADAR1 binding and editing when located distal to the guiding RNA region, but exerting an inhibitory effect when in close proximity [155].

A growing body of literature characterizes the dynamic feature of RNA post-transcriptional modifications in NSCs and neurons, such as m^6^A and m^5^C, highlighting the essential roles of the epitranscriptome in brain development. However, the regulatory mechanisms underlying cell-type or developmental-stage-specific modifications remain largely unexplored. Similarly to epigenomes, RNA chemical modifications are tightly regulated by specialized RBPs, which can be categorized into “writers,” “erasers,” and “readers”. “Writers” catalyze the installation of chemical modifications on RNA, “erasers” reverse the modified chemical groups into the original form, and “readers” recognize the modified RNAs to affect various aspects of RNA metabolism [156]. As mentioned above, m^5^C modifications exhibit notable dynamics during brain development. Researchers have analyzed m^5^C regulators in NSCs and neurons, revealing that the writer NSUN4 and the readers ALYREF and FMRP exhibit high expression levels in NSCs, while the erasers TET1 and TET3 are predominantly expressed in neurons [18]. This complementary expression pattern points to stage-specific roles of m^5^C in neurodevelopment. Future studies are needed to explore how cell-type predominant writers, erasers, and readers contribute to dynamic mRNA modifications, thereby influencing neurodevelopment.

## 5. Aberrant mRNA Processing Linked to Neurodevelopmental Disorders

Dysregulation of mRNA processing can lead to impairments in neurodevelopment and subsequent neurological disorders. Table 1 catalogs a range of NDD-associated genes that link to altered mRNA processing. For instance, RBFOX1 acts as a master splicing regulator, binding to nascent transcripts and controlling the AS of a vast network of neurologically relevant genes, including *SCN8A*, *KCNQ2*, *NLGN3*, *NLGN4X*, and *GABRB3*. The disruption of this precise splicing program due to RBFOX1 deficiency results in aberrant isoforms of these critical proteins, which underlies observed cellular defects in neuronal migration, synaptic properties, and network excitability, providing a comprehensive mechanistic basis for the disordered phenotypes associated with RBFOX1 mutations [60,157,158,159]. Similarly, PQBP1—a factor linked to microcephaly and intellectual disability—modulates both alternative splicing and polyadenylation during neurodevelopment. PQBP1 depletion perturbs the splicing of mRNAs involved in neuron projection development, resulting in impaired dendritic outgrowth. It also influences APA sites in genes regulating the cell cycle, ultimately affecting neurogenesis. Notably, disease-associated PQBP1 mutants fail to interact with splicing factors and are unable to rescue neurodevelopmental defects, underscoring the functional importance of PQBP1-mediated mRNA processing in disease etiology [125,160]. In addition, SRRM4 plays a critical role in the inclusion of neural-specific microexons (∼50 nucleotides) within genes essential for neuronal growth and synaptic function. Reduced SRRM4 levels, as observed in ASD, lead to diminished inclusion of the L microexon in the protrudin gene, compromising neurite outgrowth during neurogenesis [161]. Furthermore, a 3′-UTR variant in *FMRI* disrupts HuR binding and abolishes neuronal activity-dependent translation of FMRP, which may hinder synaptic plasticity in a clinically significant fashion [162]. In this section, we will discuss the existing literature that reports the links between mRNA processing and the pathogenesis of neurodevelopmental disorders (NDDs).

ASD, characterized by social communication deficits and repetitive behaviors, exhibits strong genetic heritability. Notably, splicing regulators such as PRP19, PTBP2, RBFOX1, and PQBP1 are recurrently mutated in ASD cohorts [60,163,164,165,166,167]. Single-nucleotide variants in these genes disrupt global splicing patterns, thereby reproducing hallmark features of ASD. In addition, the majority of AS studies in ASD patients focus on the inclusion/exclusion of microexons (3–27 nt), which are the most conserved elements of the neural splicing program. RBFOX1-mediated neuron-specific microexon 4 (24 nucleotides) of the CPEB4 gene shows a reduction in idiopathic ASD patients, leading to decreased CPEB4 protein expression. Remarkably, the imbalance in CPEB4 transcript isoforms alters the mRNA polyadenylation and protein expression of ASD-related genes and reproduces ASD-like anatomical, electrophysiological, and behavioral phenotypes [168,169]. Furthermore, recent advances in computational splicing prediction, including SpliceAI, SpliceVault, and ABSplice, have identified high-confidence splicing variants in the Spanish ASD cohort (e.g., CACNA1I, CBLB, DLGAP1, SCN2A). These variants converge on pathways regulating synaptogenesis, neurotransmission, and neuronal plasticity, reinforcing the centrality of splicing dysregulation in ASD pathogenesis [170]. Beyond splicing, 3′-UTR variants and epitranscriptomic anomalies have also been reported to be linked to ASD [171,172]. Integrative genomic analyses have identified 647 m^6^A- and 81 m^5^C-associated single-nucleotide polymorphisms (m^6^A-SNPs/m^5^C-SNPs) in ASD patients, with 38 variants overlapping the established ASD-risk genes [172]. This research offers compelling evidence regarding the interplay between RNA chemical modifications and ASD.

FXS, an inherited genetic disease that causes intellectual and developmental disabilities, arises primarily from pathogenic expansion (>200 repeats) of a CGG trinucleotide tract in the 5′-UTR of *FMR1*. This expansion silences the expression of fragile X mental retardation protein (FMRP) [173]. Intriguingly, some studies have reported a subset of FXS cases harboring pathogenic 3′-UTR variants that destabilize *FMR1* transcripts, highlighting the regulatory importance of this region [162,171,174]. Additionally, FMRP can interact with adenosine deaminases (ADAR proteins) to modulate RNA A-to-I editing [175]. Aberrant RNA editing has been discovered in FXS brains, thereby establishing a connection between RNA modification and FXS.

Rett syndrome (RTT) is a neurodevelopmental disorder characterized by a rapid regression in motor and language skills, autism-like phenotypes, ataxia, epilepsy, and progressive microcephaly [176]. The causative gene, *MECP2*, harbors multiple isoforms through alternative splicing and polyadenylation [177,178]. A number of 3′-UTR variants have been identified in Rett patients, resulting in reduced MECP2 expression in patient cell lines [179,180]. MECP2 regulates gene expression through multiple mechanisms, including transcription, microRNA processing, and mRNA splicing [181]. Early studies established a link between MECP2 and splicing regulation by demonstrating its interaction with the splicing regulator YB-1 and identifying aberrant AS patterns in an RTT mouse model [182]. Subsequent studies revealed that MECP2 also interacts with splicing factors such as LEDGF and DHX9 to regulate the splicing of glutamate receptor genes in the brain, and the altered splicing of these receptors was further linked to specific synaptic deficits in RTT model mice [183]. Studies by Sivan Osenberg et al. further revealed widespread anomalies in AS upon neuronal stimulation in RTT mice, which coincided with increased seizure susceptibility [184]. At the molecular level, MECP2 promotes the formation of higher-order biomolecular condensates with the RBFOX/LASR splicing complex. Loss of MECP2 or expression of disease-associated mutations disrupts these condensates and impairs splicing regulation [185]. These results establish MECP2 as a critical regulator of pre-mRNA splicing and illuminate how its dysfunction contributes to the pathophysiology of Rett syndrome.

ReNU syndrome is a recently discovered NDD caused by genetic variants in the noncoding gene RNU4-2, which is characterized by moderate to severe global developmental delay, ID, hypotonia, short stature, microcephaly, seizures, and distinctive dysmorphic facial features such as hooded upper eyelids, full cheeks, and a tented philtrum [186,187,188,189,190,191,192,193]. It was first identified through genetic association analyses using whole-genome sequencing data from undiagnosed NDD probands in the Genomics England 100,000 Genomes Project (100KGP) [186,189]. Recurrent variants were identified in the central region of *RNU4-2*, with Chen et al. defining an 18-base-pair critical segment (n.62–79) [189] and Greene et al. further subdividing it into two adjacent regions (n.62–70 and n.73–79) [186]. A highly recurrent de novo single-base insertion (n.64_65insT) has been detected in the majority of reported cases. The incidence of ReNU syndrome is estimated to be around 0.4% among NDDs, and it is regarded as the second most prevalent monogenic etiology for neurodevelopmental abnormality after MECP2. *RNU4-2* encodes the U4 snRNA, a key component of the major spliceosome that catalyzes pre-mRNA splicing. Variants within the critical region are hypothesized to impair U4/U6 snRNA duplex formation, compromising spliceosome activation and leading to aberrant splicing events. Such dysregulation may promote the usage of unannotated splice sites and the production of abnormal protein products, ultimately contributing to the neurodevelopmental abnormalities characteristic of the syndrome [187]. The discovery of ReNU syndrome underscores the importance of noncoding regions in genetic disorders and highlights the need for further research into the functional consequences of such variants.

**Table 1:** Alternative mRNA processing and neurological diseases.

Disease	Processing Type	Causative Genes	References
Autism spectrum disorder (ASD)	APA, AS, m^6^A, m^5^C	e.g., *HLA-G*, *AFF2*, *PRP19*, *PTBP2*, *RBFOX1*, *PQBP1*, *CPEB4*, *KIAA1671*, *INTS1*, *VSIG10*, *TJP2*, *FAM167A*, *TMEM8A*, *NUP43*	[60,163,164,165,166,167,168,169,172,175,194,195,196]
Fragile X Syndrome (FXS)	APA, AS, m^6^A, m^5^C	e.g., *FMR1*, *Mettl14*	[145,162,174]
Intellectual disability (ID)	APA, AS	e.g., *PQBP1*, *CDK5R1*	[160,197]
Rett syndrome (RTT)	APA, AS	e.g., *MECP2*	[180]
Specific Language Impairment (SLI)	APA	e.g., *ARHGEF39*	[198]
Tourette syndrome (TS)	APA	e.g., *SLITRK1*	[199]
Schizophrenia (SZ)	APA, m^6^A	e.g., *RGS4*, *EFNB2*, *CPLX2*	[200,201,202,203]
Attention-deficit hyperactivity disorder (ADHD)	APA, m^6^A	e.g., *CLOCK*, *DBH*, *MTHFR*	[203,204,205,206,207]

As our understanding of the pathogenic mechanisms underlying NDDs deepens, therapeutic strategies aimed at manipulating mRNA processing are emerging. Short antisense oligonucleotides (ASOs) offer an effective and specific way to target and alter mRNA processing in a therapeutic manner, which base-pair in an antisense orientation to a specific pre-mRNA sequence and modulate AS or APA by interfering with the protein–RNA or RNA–RNA interactions. A recent study demonstrated that ASOs effectively rescued the synaptic dysfunction caused by NRXN1 splicing defects and identified potential therapeutic targets in human brain organoids, providing novel avenues for precision therapies in ASD [208]. Furthermore, some advanced ASOs strategies have entered clinical trials for the treatment of Duchenne muscular dystrophy and spinal muscular atrophy [209,210]. Besides RNA therapeutics, chemical compounds also hold promise for clinical applications in correcting RNA-processing dysregulation [211,212]. A study using reporter-based high-throughput screening identified small molecules that broadly shift APA towards the usage of the proximal PASs, which have potential value for clinical therapeutics [212]. Collectively, these innovations underscore the therapeutic potential of precision RNA-targeting strategies for NDDs.

## 6. Conclusions and Future Prospects

In this review, we have discussed how alternative mRNA processing shapes the transcriptome to drive brain complexity and contributes to neurodevelopmental disorders. Despite these insights, several critical questions remain.

In the field of AS, despite the fact that the widespread detection of neural AS events has been propelled by technological advances in high-throughput profiling methods, the impacts of most neural AS events on neurodevelopment, such as neurogenesis, morphology, migration, and synapse formation, are largely unknown. Traditional approaches, such as generating knockout mouse models for splicing regulators, often lack the resolution to dissect isoform-specific functions. The emergence of CRISPR/Cas9 genome editing now enables the precise deletion or insertion of individual exons, offering a powerful strategy for systematically dissecting the function of neural isoforms. For instance, the NRXN gene family generates thousands of isoforms via AS at sites SS1–SS6, which are hypothesized to encode a “splicing code” for synaptic specificity. Yet, the contribution of individual NRXN isoforms to synapse type and function remains poorly understood. Future studies using CRISPR-mediated point editing to knock in or knock out specific NRXN isoforms in vivo will be instrumental in elucidating how AS regulates synaptic diversity. In addition, while many mutations in splicing factors have been implicated in neurological disorders, the underlying pathological mechanisms remain obscure, largely because these factors regulate a broad array of targets, making it difficult to link specific phenotypes to individual splicing events. Future research, such as research using brain organoids, may clarify the precise consequences of splicing factor dysfunction on brain development and function and help uncover potential treatments for splicing-related NDDs.

While there have been significant advancements in our understanding of APA regulation in recent years, the crucial issues in this field are still unresolved and require further research efforts. First, the molecular mechanisms governing developmental stage-specific APA are still incompletely characterized. Although several RBPs, such as ELAV/HU and PQBP1, have been linked to developmental switches in polyadenylation sites, systematic stage-specific screens are still needed to uncover the potential regulators and define the precise transcripts they control. Emerging proteomics and high-throughput sequencing approaches like peptide Cross-Linking and Affinity Purification (pCLAP) and single-cell long-read sequencing offer promising strategies to systematically identify candidate molecules differentially expressed at key developmental stages and to reconstruct RBP-dependent regulatory networks at single-cell resolution. These approaches will ultimately help depict a more dynamic and comprehensive picture of RNA regulation during neurodevelopment [29,213,214]. Secondly, the functional relevance of most APA events remains poorly understood. Although it is well known that proliferating cells favor the proximal PAS usage while differentiated cells prefer the distal PASs, it remains elusive whether these molecular changes are the cause or the consequence of the cell state. Novel tools like CRISPR-iPAS, a dCAS13-based APA interference system, will greatly facilitate functional studies of APA events in vivo [215]. Thirdly, how do the multiple regulatory layers (i.e., transcription, splicing, and 3′ end-processing) coordinate? Most of the well-documented mechanisms regard strategies by which the selection of AS sites and APA sites is executed locally and independently; however, accumulating evidence demonstrates extensive crosstalk between these systems. For instance, U1 snRNP, in addition to its splicing role, prevents intronic polyadenylation at cryptic PASs by inhibiting the assembly of 3′ end-processing complex [216]. Future mechanistic studies must elucidate how these processing steps are temporally and spatially integrated to ensure transcriptome fidelity.

While significant advancements have been achieved in mapping m^6^A, m^5^C, and other mRNA modification patterns during brain development, the site-specific functions of these marks remain incompletely elucidated. The emergence of CRISPR–Cas13-based RNA editing technologies offers a precise means to selectively alter individual methylated sites, enabling an in vivo functional assessment of particular modifications. By integrating multi-omics methodologies such as m^6^A sequencing, RNA bisulfite sequencing, and proteomic profiling, researchers can decode the “epitranscriptomic code” governing neuronal identity and maturation. Moreover, combining live-cell imaging of dynamic methylation with computational modeling will shed light on how transient RNA modification events translate into long-term changes in neuronal connectivity.

In conclusion, addressing these questions will deepen our understanding of the gene regulatory networks that sculpt neurodevelopment and the etiology of neurodevelopmental disorders, paving the way for new therapeutic avenues.

## Data Availability

No new data were created or analyzed in this study.

## Abbreviations

The following abbreviations are used in this manuscript:

A3SS	alternative 3′ splice sites
A5SS	alternative 5′ splice sites
ADAR	adenosine deaminase RNA-specific
ALYREF	Aly/REF export factor
APA	alternative polyadenylation
AS	alternative splicing
ASD	autism spectrum disorder
ASOs	antisense oligonucleotides
BAF	BRG1/BRM-associated factor
BDNF	brain-derived neurotrophic factor
CALM1	calmodulin 1
CBLB	Casitas B-lineage lymphoma proto-oncogene b
CDS	coding sequence
CFIm	cleavage factor I m
CFIIm	cleavage factor II m
CNS	central nervous system
CPA	cleavage and polyadenylation
CPEB4	cytoplasmic polyadenylation element-binding protein 4
CPSF	cleavage and polyadenylation specificity factor
CR-APA	coding-region APA
CRISPR-iPAS	CRISPR interference of polyadenylation sites
CstF	cleavage stimulation factor
CstF64τ	CstF64 paralog tau
DLGAP1	DLG-associated protein 1
DPF2	double PHD fingers 2
DRG	dorsal root ganglion
ELAVL	ELAV-like neuron-specific RNA binding protein
FMRP	fragile X mental retardation protein
FMR1	fragile X messenger ribonucleoprotein 1
m1A	N1-methyladenosine
m5C	5-methylcytosine
m6A	N6-methyladenosine
m7G	N7-methylguanosine
MECP2	methyl-CpG-binding protein 2
MXE	mutually exclusive exons
NDDs	neurodevelopmental disorders
Nm	2′-O-methylation
NOVA	neuro-oncological ventral antigens
NPCs	neural progenitor cells
NSCs	neural stem cells
NSPCs	neural stem/progenitor cells
NSUN2	NOP2/Sun RNA methyltransferase 2
NSUN4	NOP2/Sun RNA methyltransferase 4
NMD	nonsense-mediated mRNA decay
NRXNs	neurexins
PAP	poly(A) polymerase
PASs	polyadenylation sites
pCLAP	peptide Cross-Linking and Affinity Purification
PQBP1	polyglutamine-binding protein 1
PRP19	pre-mRNA processing factor 19
PTBP	polypyrimidine-tract-binding protein
RBFOX	RNA-binding fox-1 homolog
RI	retained introns
RNAP II	RNA polymerase II
RTT	Rett syndrome
ScISOr-Seq2	Single-cell Isoform Sequencing 2
SCN2A	sodium voltage-gated channel alpha subunit 2
SE	skipped exons
SHTN1	shootin 1
SRRM4	serine/arginine repetitive matrix 4
TET	tet methylcytosine dioxygenase
USE	upstream sequence element
UTR-APA	3′-untranslated-region APA
